# First-principles insights into the electronic structure, optical and band alignment properties of earth-abundant Cu_2_SrSnS_4_ solar absorber

**DOI:** 10.1038/s41598-021-84037-8

**Published:** 2021-02-26

**Authors:** Nelson Y. Dzade

**Affiliations:** Cardiff University, Main Building, Park Place, Cardiff, CF10 3AT UK

**Keywords:** Materials for devices, Theory and computation, Solar cells

## Abstract

Cu_2_SrSnS_4_ (CSTS) is a promising alternative candidate to Cu_2_ZnSnS_4_ (CZTS) for single- or multi-junction photovoltaics (PVs) owing to its efficient light-absorbing capability, earth-abundant, nontoxic constituents, and suitable defect properties. However, as a novel absorber material, several fundamental properties need to be characterized before further progress can be made in CSTS photovoltaics. In this letter, hybrid density functional theory (DFT) calculations have been used to comprehensively characterize for the first time, the electronic structure, band alignment, and optical properties of CSTS. It is demonstrated that CSTS possesses the ideal electronic structure (direct band gap of 1.98 eV and small photocarrier effective masses) and optical properties (high extinction coefficient and wide absorption) suitable for photovoltaic applications. Simulated X-ray photoelectron spectroscopy (XPS) valence band spectra using variable excitation energies show that Cu-3*d* electronic state dominates the valence band maximum of CSTS. Furthermore, the vacuum-aligned band diagram between CSTS and other common absorbers (CZTS, CIGS, CdTe) and the common *n*-type partner materials (CdS, ZnO) was constructed, which indicate staggered type-II band alignment at the CSTS/CdS and CSTS/ZnO interfaces. Based on these results, interface band offset engineering and alternative device architectures are suggested to improve charge carrier separation and power conversion efficiencies of CSTS.

## Introduction

Material utilization is one of the most critical considerations in determining the manufacturing cost of solar cells. Photovoltaic (PV) technology can only provide a significant fraction of the world’s energy demands if solar devices are composed of earth-abundant and nontoxic materials^1^. Copper indium gallium selenide (CIGS) and cadmium telluride (CdTe) solar cells have already attained impressive 10–25% solar conversion efficiencies^2–5^, but the scarcity and toxicity associated with the In, Ga, Cd, and Te elements present in these cells limit their sustainability in the future^6^. Among the alternative absorber materials to replace CIGS and CdTe for thin-film solar cells, kesterite materials (Cu_2_ZnSn(S,Se)_4_ (CZTSSe)) have recently emerged as promising candidates because they combine (i) near optimum direct bandgaps (1.5 eV for CZTS and 1.13 eV for CZTSSe), (ii) high optical absorption coefficient of ~ 10^4^ cm^−1^ in the visible light region, with predicted theoretical power conversion efficiency (PCE) more than 30%, and (iii) constituent elements that are earth-abundant, cheap, and non-toxic^7–12^.

Despite their superior optoelectronic properties, the highest reported efficiency for CZTS-based solar devices has been stagnated at around 12.6% for CZTSSe^9,13^. The efficiency stagnation has been attributed in part to significant CuZn and ZnCu antisite disordering in CZTS/Se materials. In the CZTSSe compounds, Cu, Zn, and Sn cations have similar tetrahedral coordination and comparable ionic radii (Cu^+^ (0.91 Å), Zn^2+^ (0.88 Å), and Sn^4+^ (0.83 Å)). These similarities contribute to small antisite defect formation energies and their prevalence in CZTS/Se materials^14^. The antisite defects cause potential fluctuations in the electronic band structure and band tailing, which in turn effectively limits the open-circuit voltage (V_oc_) of fabricated devices^15–17^. Besides, CuSn, SnCu, ZnSn, and SnZn antisite defects can contribute deep level defect states within the bandgap of CZTS/Se materials, thus increasing charge carrier recombination rates.

One effective strategy to suppress the antisite disorder in kesterite materials is to replace the Zn ion with a much larger alkaline earth cation such as Ba or Sr, which introduces structural and ionic size diversity into the lattice^18^. The resulting Cu_2_BaSnS_4_ (CBTS) and Cu_2_SrSnS_4_ (CSTS) compounds exhibit better defect properties than kesterite Cu_2_ZnSnS_4_ (CZTS) because of the larger size of Ba^2+^ (1.49 Å) and Sr^2+^ (1.32 Å) and the lower structural symmetry, which can suppress the formation of antisite defects^19^. CSTS compound is of considerable interest for photovoltaics owing to its sharp band edges and suitable band gap (1.78–2.1 eV) for a top absorber in tandem cells. CSTS has trigonal crystal structure^20–23^, which features CuS_4_ and SnS_4_ tetrahedra that share common corners, forming a 3D network analogous to kesterite. However, unlike the kesterite structure, the large electropositive cations Sr^2+^/Ba^2+^ sit inside an S_8_ square antiprism. The differences in size and the coordination environments around the Cu^+^, Sr^2+^/Ba^2+^, and Sn^4+^ should discourage the formation of Cu–Sr/Ba and Sn–Sr/Ba antisite disorder. Previous theoretical studies of the optoelectronic and defect properties of Cu_2_-II-SnS_4_ (II = Ba, Sr) with the P3_1_ phase, predicted a direct bandgap of 1.79 (1.78) eV for CBTS (CSTS)^19^. Recent first-principles calculations of the phonon dispersion of CSTS and CBTS show that the compounds are stable at ambient pressure^24^. However, there is no systematic theoretical investigation dedicated to elucidating the structural, optoelectronic, and interface band alignment properties of these compounds, which makes this investigation timely.

Herein, a comprehensive hybrid density functional theory (DFT) description of the electronic structure, energy-dependent X-ray photoelectron spectra (XPS) valence band spectra at variable excitation energies, the optical, surface, and interface properties of CSTS are presented. CSTS is demonstrated to possess a direct bandgap of 1.98 eV, a high extinction coefficient, and broad absorption, suggesting that CSTS can harvest a larger fraction of the solar spectrum when used as an active absorber layer even in ultrathin film. Analysis of the simulated valence band photoemission results provide strong evidence that Cu-3*d* electronic states dominate the valence band maximum of CSTS. Lastly, the structure and properties of the most stable (100) and (110) surfaces of CSTS were systematically characterized, and the fundamental energy band alignment between CSTS and other common absorbers (CZTS, CIGS, CdTe) and common n-type partner materials (CdS, ZnO) is constructed and discussed.

## Material and methods

### Computational details

The first-principles density functional theory calculations were carried out within the VASP -Vienna Ab initio Simulation Package^25,26^. The interactions between the valence and core electrons was described using projected augmented wave (PAW) method^27^. Geometry optimizations were performed using the generalized gradient approximation PBE (Perdew–Burke–Ernzerhof) functional^28^. The electronic structure and optical properties were predicted using the screened hybrid functional HSE06^29^ with a 25% Hartree–Fock exchange. A plane-wave energy cut-off of 600 eV and Monkhorst-Pack^30^ K-points mesh of 7 × 7 × 3 was used to converge the total energy of the CSTS to within 10^−6^ eV and the residual forces on all relaxed atoms to 10^−3^ eVÅ^−1^.

The electrostatic potential of the surfaces was averaged along the c-direction, using the MacroDensity package^31,32^. A 20 Å vacuum region was added the c-direction to avoid any spurious interactions between periodic slabs. By aligning the slab vacuum level to the bulk core-level eigenvalues in the center of the slab, using S 1s core level as reference state, the ionization potential (IP) can be determined. The work function (Φ) is calculated as: Φ = E_v_−E_F_, where E_v_ and E_F_ are the vacuum and the Fermi level, respectively. In all calculations, dipole correction perpendicular to the surfaces was accounted for. The XPS valence band spectra were simulated using the GALORE code^33^, a materials design tool for transforming ab-initio partial density of states (PDOS) to XPS spectra by employing the Gelius method^34,35^ to apply weightings to the atom PDOS using photoionization cross-sections from reference data.

## Results and discussion

### Crystal structure

**Figure 1:**
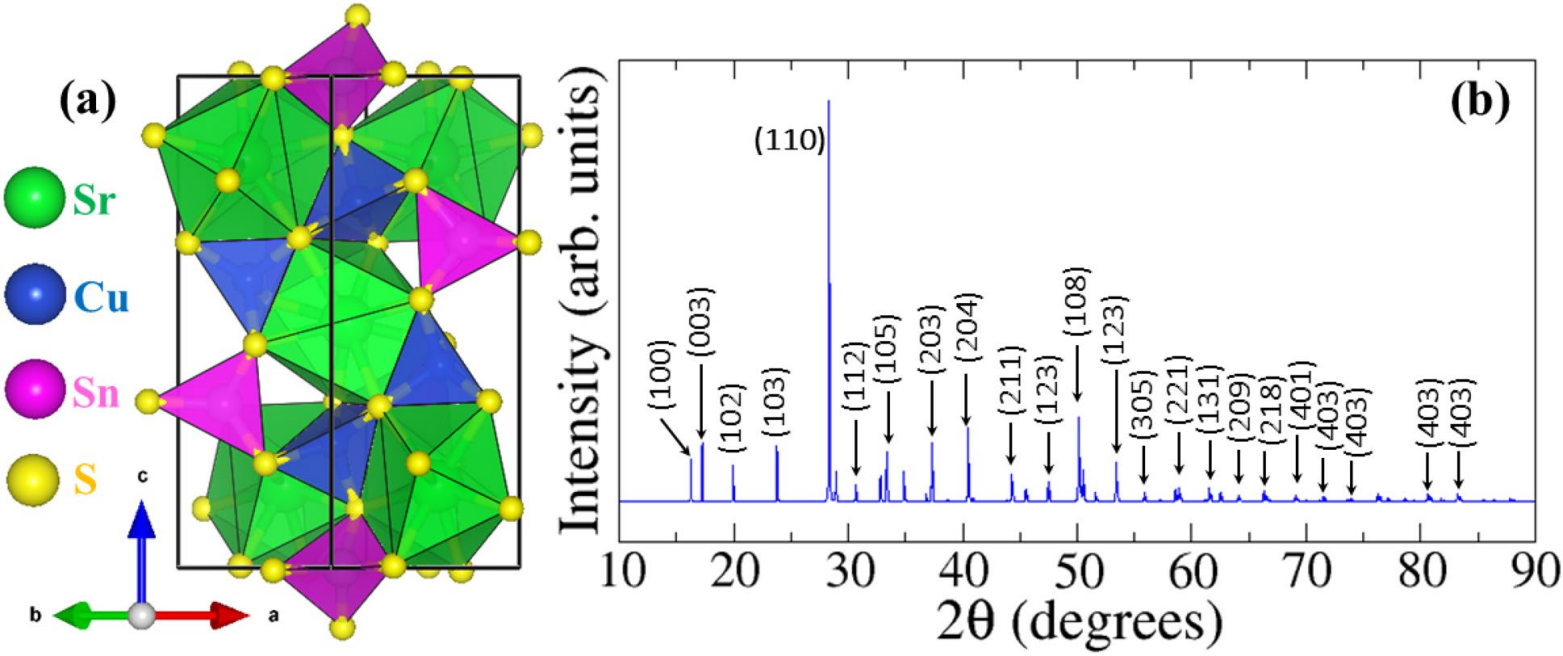
(**a**) Trigonal crystal structure produced by VESTA-ver.3.5.7^38^ and (**b**) the corresponding simulated powder diffraction pattern of Cu_2_SrSnS_4_.

CSTS adopts the trigonal space group P3_1_^20^, with lattice parameters *a* = 6.25 Å, *c* = 15.57 Å (Fig. 1a). The trigonal structure features CuS_4_ and SnS_4_ tetrahedra that share common corners, forming a 3D network analogous to kesterite. But unlike the kesterite structure, the Sr^2+^ cation sits inside an S_8_ square antiprism. The primitive unit cell contains 24 atoms (3 Sr, 6 Cu, 3 Sn, and 12 S atoms). A full unit cell relaxation yielded a strain-free CSTS with lattice parameters *a* = 6.300 Å, and *c* = 15.495 Å, which compares closely with experimental^20^ and previous theoretical^19,36,37^ results. The average Sr–S, Cu–S, and Sn–S relaxed bond distances are predicted at 2.309, 3.092, and 2.427 Å, respectively. Based on the optimized structure, the powder diffraction pattern of CSTS is simulated using the VESTA Crystallographic software^38^ as shown in Fig. 1b. All assigned peaks in the simulated DFT spectrum match very closely with the experimental X-ray Diffraction (XRD) patterns of Crovetto et al.^23^.

### Electronic band structure and related properties

**Figure 2:**
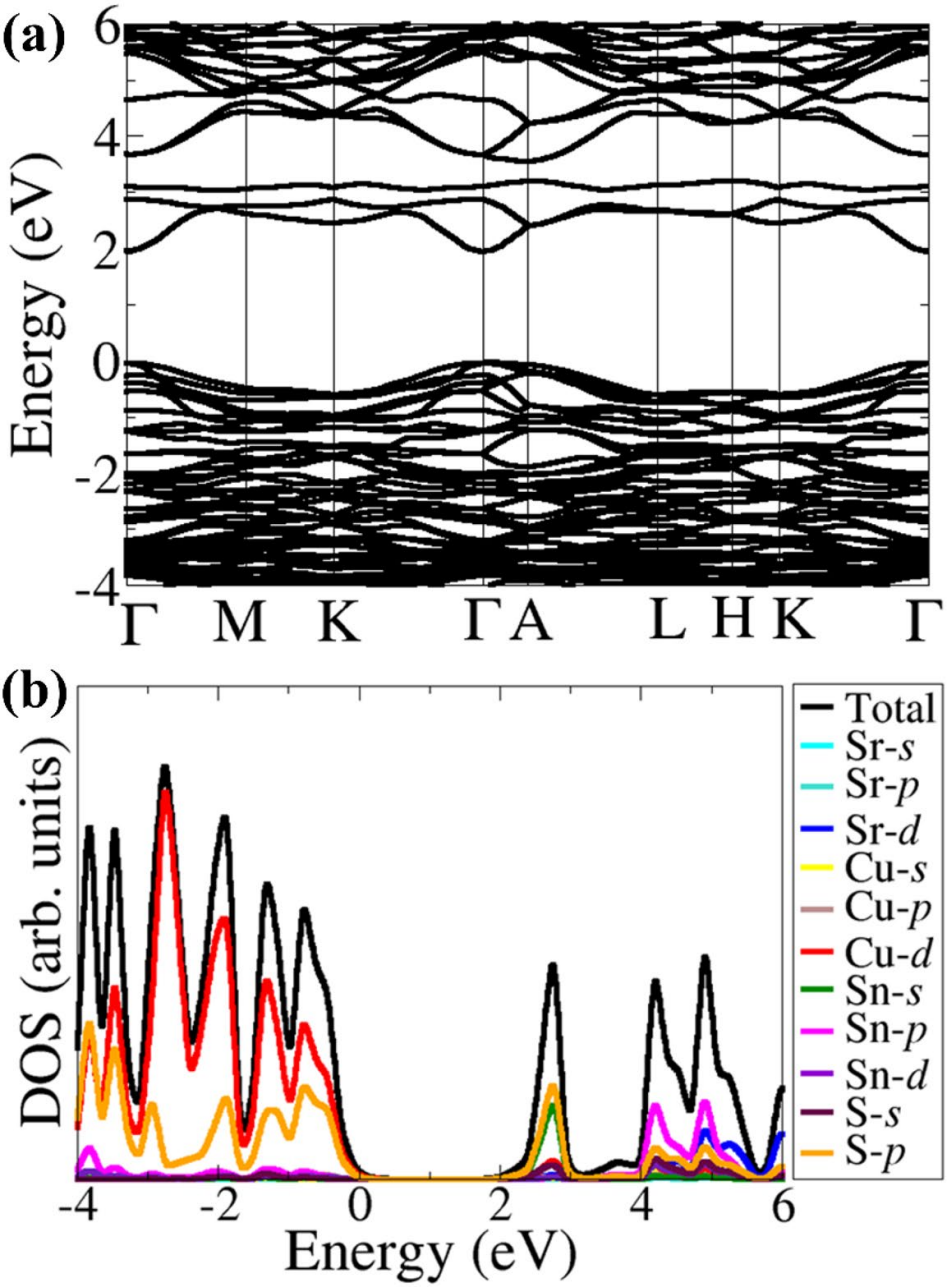
(**a**) band structure along the high-symmetry directions and (**b**) projected electronic density of states of Cu_2_SrSnS_4_ obtained using HSE06 functional.

The band structure (Fig. 2a) shows that CSTS is a direct bandgap material at the Brillouin zone’s gamma-point. The bandgap is predicted at 1.98 eV, which is in good agreement with known experimental (1.78–2.1 eV)^21–23^ and previous DFT predictions^19,37,38^. From the predicted band structure, the effective masses of electrons (${m}_{e}^{*}$) and holes (${m}_{h}^{*}$) were calculated by fitting the energy of the conduction band minimum and valence band maximum, respectively, to a quadratic polynomial in the reciprocal lattice vector *k*: ${m}_{e\left(h\right)}^{*}=\pm {\hslash }^{2}{\left(\frac{{d}^{2}{E}_{k}}{d{k}^{2}}\right)}^{-1}$. The effective masses are intricately linked to the diffusion coefficient ($D = \frac{{k_{B} T}}{e}\mu$) and mobility ($\mu = e\frac{\tau }{{m^{*} }}$) of the charge carriers in a semiconductor. Generally, small effective masses give rise to higher charge carrier mobility and diffusion^39^. Summarized in Table 1 are the calculated ${m}_{e}^{*}$ and ${m}_{h}^{*}$ in selected directions of the Brillouin zone. The smallest ${m}_{e }^{*}$ appears in the direction from Γ–A (0.0013), whereas the highest appears in the M–K (0.1395) direction. The effective hole masses are also generally small due to the reduced dispersion of the VBM, indicating that holes should be mobile in CSTS. The predicted small effective masses are highly desirable for promoting efficient separation of photogenerated charge carriers in CSTS, which is vital for fabricating high-efficiency solar devices. The recombination rate of charge carriers in CSTS is ascertained by calculating the hole to electron effective mass ratio (*D* = m^∗^_h_/m^∗^_e_)^40^. The larger *D* values, particularly along the Γ–M (11.6897), K–Γ (6.4074), Γ–A (4.6923), and A–L (4.0000) directions on the Brillion zone points to efficient separation and low recombination of photogenerated charge carriers in CSTS^41,42^.

**Table 1:** Effective masses of holes (valence band maximum) and electrons (conduction band minimum) of Cu_2_SrSnS_4_ along high symmetry directions.

Direction	m^∗^_h_ (m_e_)	m^∗^_e_ (m_e_)	D = m^∗^_h_/m^∗^_e_
Γ–M	0.0339	0.0029	11.6897
M–K	0.0130	0.1395	0.0932
K–Γ	0.0173	0.0027	6.4074
Γ–A	0.0061	0.0013	4.6923
A–L	0.0248	0.0062	4.000
L–H	0.0159	0.1292	0.1231
H–K	0.0039	0.0042	0.9286

### Simulated XPS valence band spectra

**Figure 3:**
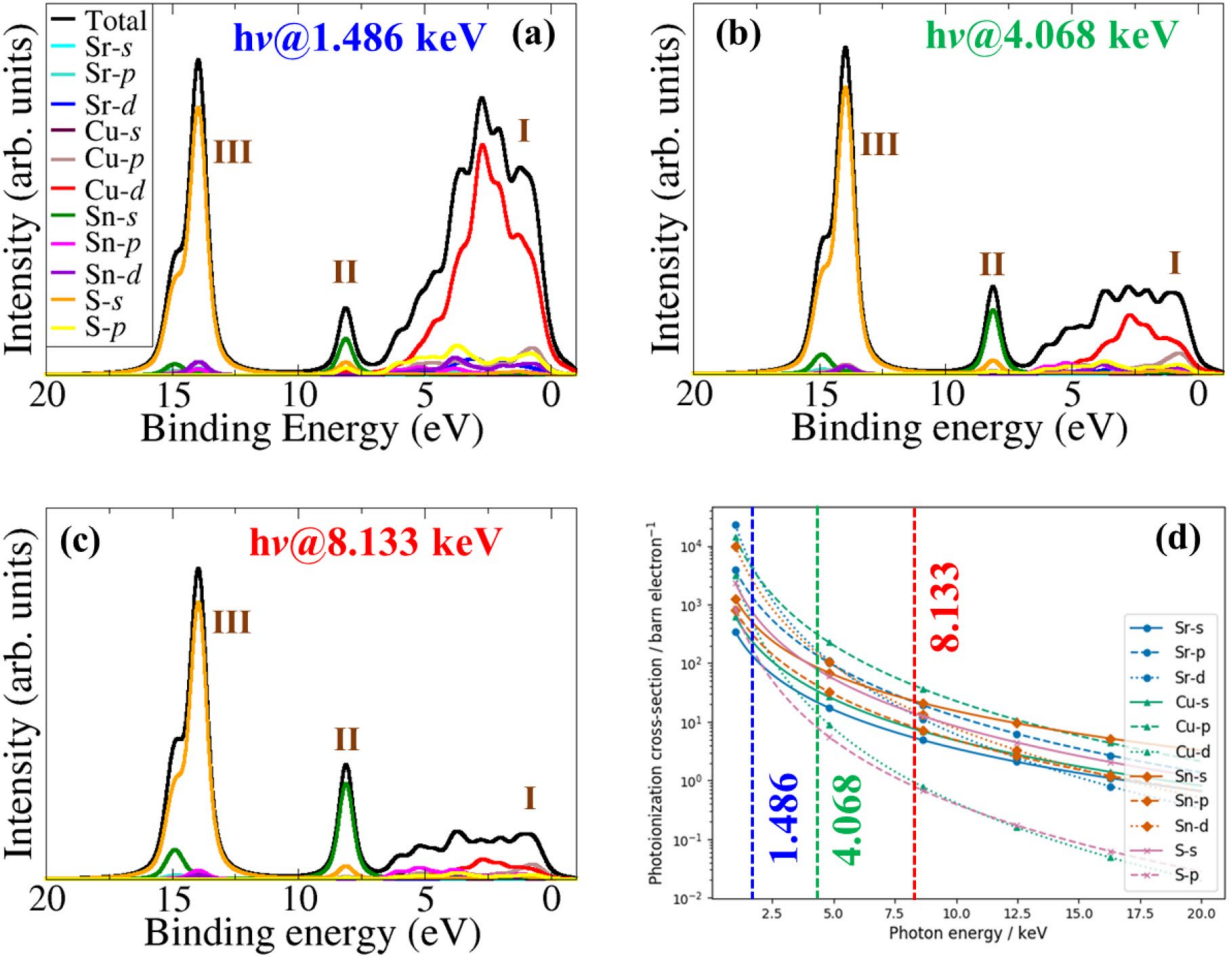
Simulated valence band edge spectra of Cu_2_SrSnS_4_ at (**a**) Al Kα_1_ (hν = 1.486 keV), (**b**) hν = 4.068 keV and (**c**) hν = 8.133 keV. The photoionization cross-section dependence on the ionizing photon energy for the valence orbitals in Cu_2_SrSnS_4_ is shown in (**d**).

The XPS valence band spectra of a semiconductor are interpreted by direct comparison with DFT calculated projected density of states (PDOS) weighted by the photoionization cross-sections, and good agreement is generally observed^43–47^. The non-weighted of PDOS of CSTS (Fig. 2b) shows that the valence band maximum (VBM) is dominated by Cu-3*d* and S-2*p* states, whereas the conduction band minimum (CBM) is dominated by S-3*p* and Sn-5s, with small contributions from Cu-3*d* and S-1*s* states. The photoionization cross-section depends on the probe radiation, the orbital shapes, and their energies. To account for this, the GALORE software^33^ employs the Gelius method^34,35^ to apply weightings to the atom projected PDOS using the photoionization cross-sections formulated by Scofield^48^. The simulated valence band photoelectron spectrum of CSTS at Kα1 (1486 eV), as well as at 4068 eV and 8133 eV photon energies (*i.e.*, soft to hard X-ray ionization photon energy (hν)) are shown in Fig. 3. The main valence band has a spectral onset from 0.0 eV and expands up to 7 eV in binding energy. Besides, broad peaks are found on the high-binding-energy side of the main valence band, centered at 8 eV and 14 eV binding energy. Three spectral features can be assigned: I for the main valence band region and II and III for the broad satellite features at 8 eV and 14 eV. The intensity of I relative to II and III continuously increases while moving from soft to hard X-ray ionization photon energy (hν), resulting in a change in the valence band spectrum’s shape. At the same time, switching from soft to hard X-ray hν leads to a marked increase in the broad satellite features’ relative intensity at 8 eV and 14 eV high binding energy sides. The observed changes in the valence band spectral feature originate from the hν-dependence of the photoionization cross-section of the atomic orbitals contributing to the valence levels. Due to their higher photoionization cross-section (Fig. 3d), Cu-3*d* photoelectrons dominate the main valence band region (I) for CSTS. The high binding energy satellite broad feature at 8 eV can be assigned mainly to the contribution from Sn-5*s*, whereas the broad feature at 14 eV can be assigned to contributions mainly from S-3*s*. The Cu-3*d* photoionization cross-section decreases much rapidly than Cu-3*p* and S-3*p* with photon energy, so at higher photon energies, its contribution becomes less significant (Fig. 3a–c). Although there are no available experimental XPS data to directly compare with the simulated XPS spectra, the simulated XPS spectra at different excitation energies may help clarify future experiments. The results can also help assign the atomic orbital contributions to various features (peaks) observed in X-ray photoelectron spectroscopy measurements.

### Optical properties

**Figure 4:**
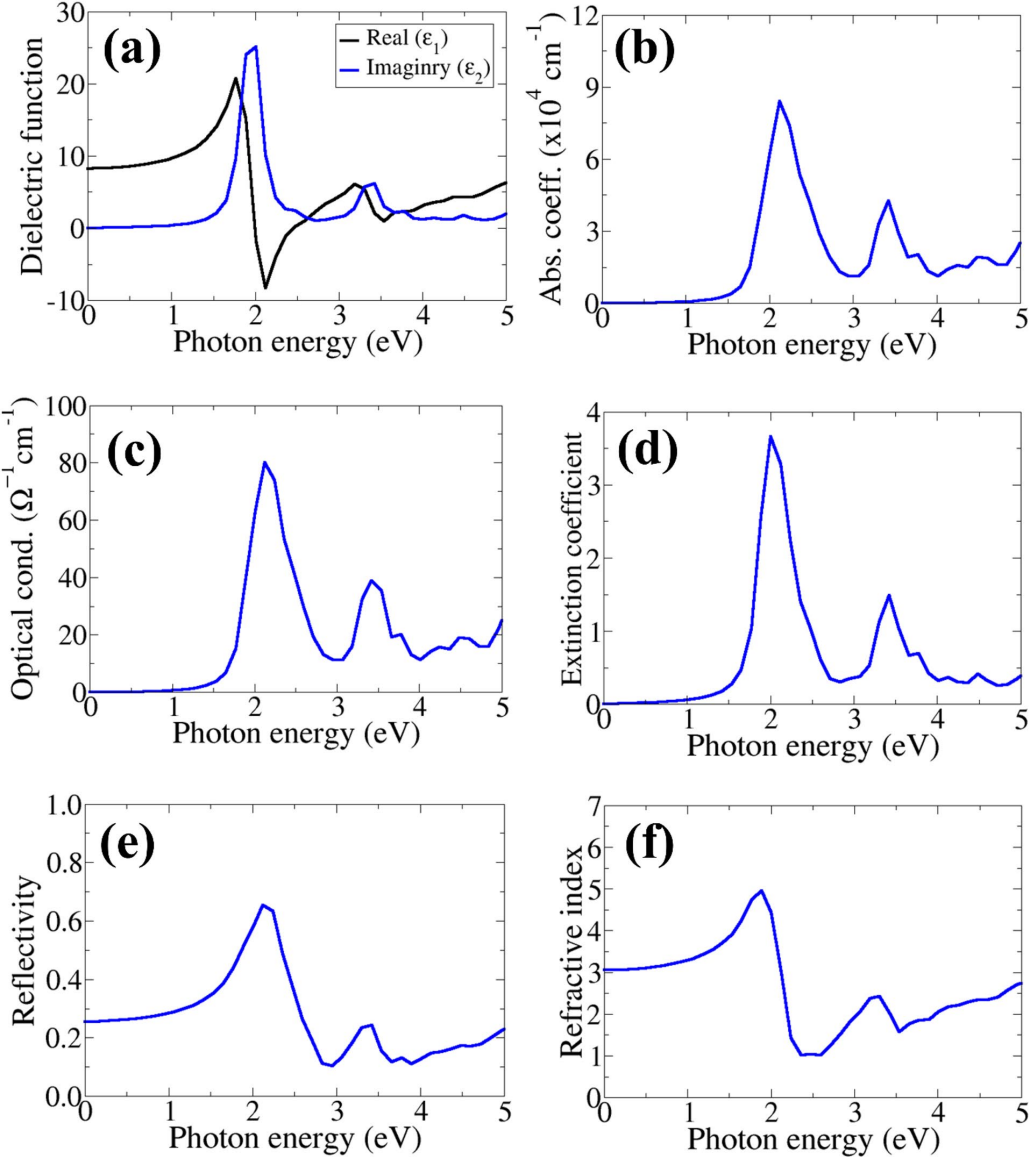
Calculated (**a**) real and imaginary part of the dielectric function, (**b**) absorption, (**c**) optical conductivity, (**d**) extinction coefficient, (**e**) reflectivity, and (**f**) refractive index of Cu_2_SrSnS_4_.

The optical properties, including dielectric function, optical absorption and conductivity, reflectivity, refractive index, and the extinction coefficient for CSTS have been characterized for the photon energy from 0 to 10 eV. The calculated real (dispersive, ε_1_) and imaginary (absorptive, ε_2_) parts of the dielectric function for CSTS are shown in Fig. 4a. The results show that CSTS has a higher dielectric constant at low energy region (from 0 to 2 eV) with the onset dielectric constant predicted at 8.25 (Fig. 4a), which compares closely to that of Cu_2_ZnSnSe_4_ reported at 8.64. A high dielectric constant is a desired property for potential photovoltaic materials as it indicates an increased ability to screen charge and decrease charge carrier recombination rates^49–54^. Therefore, the high dielectric constant predicted for CSTS makes it an attractive candidate to achieve enhanced photovoltaic performance. The combination of large dielectric constants and low effective masses calculated from the electronic band structure is expected to promote effective ionization of defect states^55^, leading to fewer deep trap states in CSTS. It can be seen from the absorption spectra (Fig. 4b) that the absorption starts at around 1.98 eV, which corresponds to the fundamental bandgap. CSTS exhibits a high absorption coefficient in the order of 10^4^ cm^−1^ in the visible light region, which makes it a suitable material for the photovoltaic application. For completeness of the optical properties, the optical conductivity (Fig. 4c), extinction coefficient (Fig. 4d), reflectivity (Fig. 4e), and refractive index (Fig. 4f) for CSTS are calculated. The optical conductivity is a good gauge of photoconductivity that could shed light on the electrical conductivity of the material^56,57^. The features of the optical conductivity are quite identical with that of the absorption spectra, with the highest absorption and optical conductivity predicted at 2.1 eV. The low reflectivity (~ 25%) and high extinction coefficient (~ 3.7) for CSTS also show that this material is ideal for photovoltaic and optoelectronic applications. The high extinction coefficient and wide absorption for CSTS indicate that it can harvest a larger fraction of the solar spectrum when used as an active absorber layer even in ultrathin film. The low refractive index of 3.05 for CSTS, comparable to the refractive index of Si (3.4 at 550 nm)^58^ and CdTe (~ 3.0 at 600 nm)^59^, is also ideal for photovoltaic application. When compared with perovskite materials, the refractive index of CSTS if higher than that of CH_3_NH_3_PbI_3_ (2.61 at 633 nm)^60^ and CsPbBr_3_ (1.85–2.30 at 400–530 nm)^61^.

### Surface and interface properties

**Figure 5:**
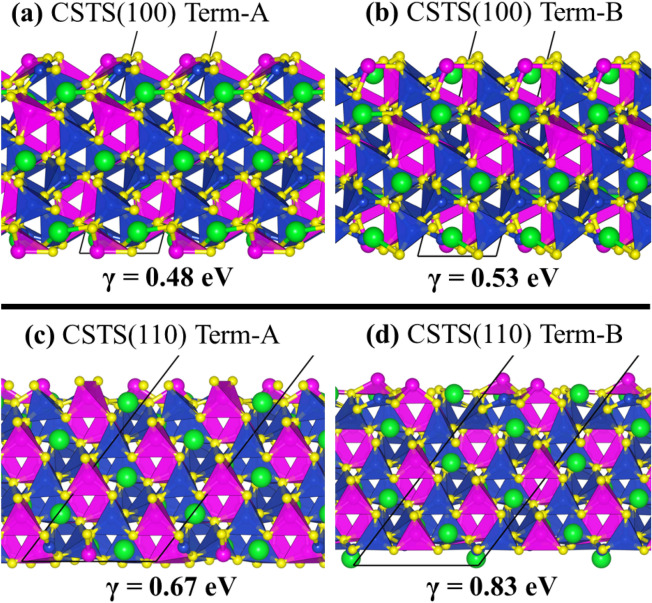
Side views of the relaxed structures of the (100) and (110) surfaces of Cu_2_SrSnS_4_. Images produced using VESTA-ver.3.5.7.^38^.

**Figure 6:**
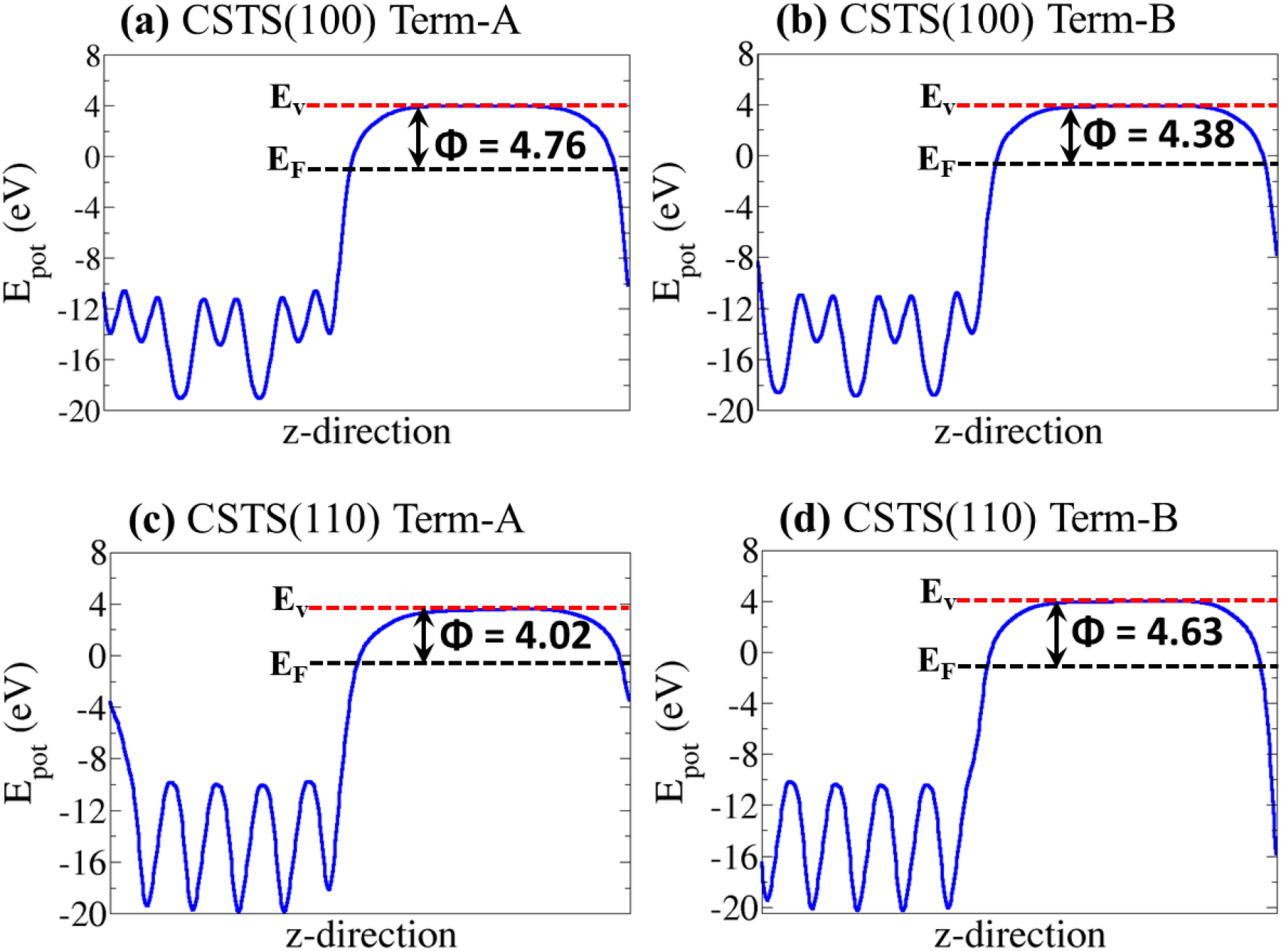
The electrostatic potentials (blue continuous line) for the (100) and (110) surfaces of Cu_2_SrSnS_4_ in Termination-A and Termination-B. The red and black dashed lines represent the vacuum level (Ev) and the Fermi level (E_F_), respectively. The Φ is the work function.

As a new solar absorber material, studies dedicated to the characterisation of the surface structure, composition, and relative stabilities of the major surfaces of CSTS are limited. Evaluation of the surface and interfacial properties of CSTS would ultimately dictate its suitability as a thin film solar absorber material. Therefore, the structure and properties of the two most stable (100) and (110) surfaces of CSTS have been systematically characterized. Each surface has two non-dipolar terminations (denoted as termination A and B), both of which were characterized to determine the most stable one. Shown in Fig. 5 are the relaxed structures of the (100) and (110) surfaces of CSTS, with the surface energy of the most stable termination-A calculated at 0.48 and 0.67 Jm^−2^, respectively, (see Table 2). These results suggest that the (100) is thermodynamically more stable than the (110) surface. The work function (Φ) of termination A (B) of the CSTS (100) is calculated at 4.76 (4.38 eV) vs. vacuum as shown in Fig. 6a,b. For the CSTS (110) surface, the Φ of termination A (B) is 4.02 (4.63 eV), as shown in Fig. 6c,d.

**Table 2:** Calculated surface energy (γ), vacuum level (*E*_vac_), Fermi level (*E*_F_), work function (Φ), Ionization potential (IP), and electron affinity of the (100) and (110) surfaces of Cu_2_SrSnS_4_.

Parameter	CSTS(100)		CSTS(110)	
	Termination-A	Termination-B	Termination-A	Termination-B
γ (Jm^─2^)	0.48	0.53	0.67	0.83
Φ (eV)	4.76	4.38	4.02	4.63
IP (eV)	5.50	5.26	5.30	5.31
EA (eV)	3.48	3.24	3.28	3.29

**Figure 7:**
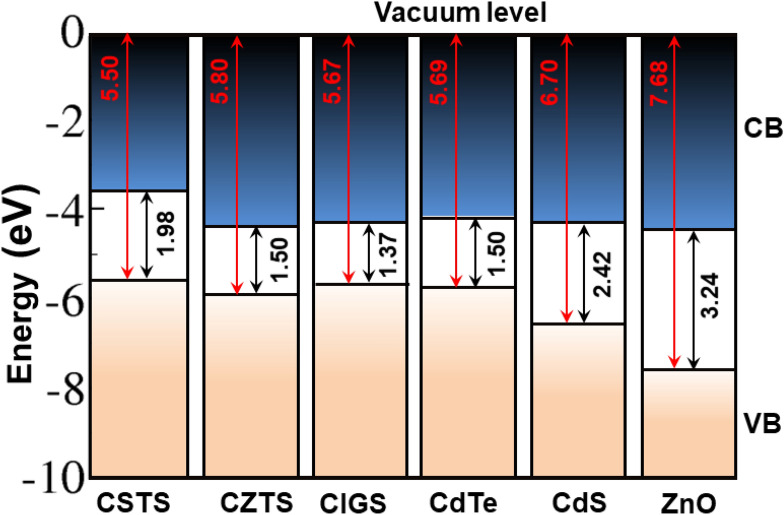
Vacuum-aligned band diagram between CSTS and other common absorbers (CZTS, CIGS, CdTe) and the common n-type partner materials (CdS, ZnO). Literature values of IP and band gap are taken for CZTS^62^, CIGS^63^, CdTe^64^, CdS^62^, and ZnO^65^.

Using the most stable CSTS (100) surface, the band alignment of CSTS is shown in Fig. 7. The valence band energy (ionization potential, IP) of CSTS (100) was found to be 5.50 eV below the vacuum level, and is noticeably very similar to other commonly used solar absorbers [CZTS (5.80 eV)^62^, CIGS (5.67)^63^, and CdTe (5.69 eV)^64^]. The band alignments for common *n*-type partner materials (CdS and ZnO) is also shown in Fig. 7. Considering that the magnitude of the band offset at the absorber/buffer interface controls transport phenomena and characteristics of fabricated devices, an accurate determination of band offsets at semiconductor heterojunctions interfaces is also of great interest. From Fig. 7, a staggered type-II band alignment is predicted to exist between the valence and conduction bands at the CSTS/CdS and CSTS/ZnO interfaces. The conduction band offset (CBO) and the valence band offset (VBO) at the CSTS/CdS interface are calculated at 0.76 and 1.20 eV. At the CSTS/ZnO, the CBO and VBO are estimated at 0.92 and 2.18 eV, respectively. These results suggest that CdS would be a more appropriate choice of partner material for CSTS from an electronic point of view, because of the smaller CBO between CSTS and CdS, which is also often the first successful choice as the n-type partner material for CIGS^66^, CdTe^67^, and CZTS^68,69^ absorbers. The slightly larger CBO at the CSTS/CdS interface compared to that of the CZTS/CdS interface (0.21 eV)^68,69^, indicates that interface and band offset engineering is required to lower the barrier height at the CSTS/CdS interface and promote efficient charge carrier separation. As was demonstrated to improve the performance of SnS solar cells^70,71^, a contact metal with lower work function may be needed at the opposite sides of the junction for CSTS.

## Summary and conclusions

In summary, the electronic structure, optical properties, and energy band alignment of CSTS have been systematically characterized for the first time employing hybrid density functional theory calculations. It is demonstrated CSTS possesses appropriate electronic structures and optical properties for solar energy applications. Analysis of the simulated valence band photoemission results provides strong evidence that Cu-3*d* electronic states dominate the valence band maximum of CSTS. A staggered type-II band alignment is predicted at the CSTS/CdS and CSTS/ZnO interfaces, with CdS giving a smaller conduction band offset than ZnO. These results suggest that CSTS is a suitable earth-abundant and low-cost absorber material for efficient solar device fabrication although further investigations of interface and band offset engineering to improve charge carrier separation will be needed in the near future.

## Data Availability

Information on the data that underpins the results presented here, including how to access them, can be found in the Cardiff University data catalogue at 10.17035/d.2021.0128072759.
